# 1,3-Dimethyl-1*H*-1,2,3-benzotriazol-3-ium tetra­chloridoferrate(III)

**DOI:** 10.1107/S1600536813003711

**Published:** 2013-02-13

**Authors:** Zan Sun, Dong-Cheng Hu, Jia-Cheng Liu

**Affiliations:** a Key Laboratory of Eco-Environment-Related Polymer Materials of the Ministry of Education, Key Laboratory of Polymer Materials of Gansu Province, Key Laboratory of Bioelectrochemistry & Environmental Analysis of Gansu Province, College of Chemistry and Chemical Engineering, Northwest Normal University, Lanzhou 730070, People’s Republic of China

## Abstract

The asymmetric unit of the title salt, (C_8_H_10_N_3_)[FeCl_4_], contains one 1,3-dimethyl-1*H*-1,2,3-benzotriazol-3-ium cation and one tetra­chloridoferrate anion. The Fe^III^ atom in the anion is tetra­hedrally coordinated by four Cl atoms. In the crystal, inter­actions are observed between the Cl atoms and the triazolium ring [Cl⋯centroid distances = 3.587 (3) and 3.866 (3) Å].

## Related literature

For related iron complexes, see: Hay *et al.* (2003 ▶); Liu *et al.* (2000 ▶); Lorenz *et al.* (2000 ▶); Shapley *et al.* (2003 ▶).
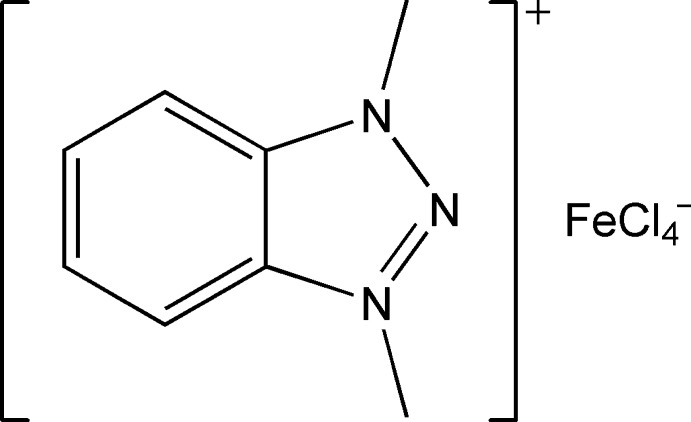


## Experimental

### 

#### Crystal data

(C_8_H_10_N_3_)[FeCl_4_]
*M* *_r_* = 345.84
Orthorhombic, 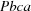

*a* = 10.2920 (5) Å
*b* = 12.5518 (6) Å
*c* = 22.5857 (9) Å
*V* = 2917.7 (2) Å^3^
*Z* = 8
Mo *K*α radiation
μ = 1.74 mm^−1^
*T* = 293 K
0.32 × 0.28 × 0.25 mm

#### Data collection

Oxford Diffraction SuperNova (Dual, Cu at zero, Eos) diffractometer
Absorption correction: multi-scan (*CrysAlis PRO*; Oxford Diffraction, 2012 ▶) *T*_min_ = 0.578, *T*_max_ = 0.647
8114 measured reflections
3016 independent reflections
1828 reflections with *I* > 2σ(*I*)
*R*_int_ = 0.031

#### Refinement

*R*[*F* ^2^ > 2σ(*F* ^2^)] = 0.057
*wR*(*F* ^2^) = 0.183
*S* = 1.05
3016 reflections
147 parameters
H-atom parameters constrained
Δρ_max_ = 0.53 e Å^−3^
Δρ_min_ = −0.39 e Å^−3^

### 

Data collection: *CrysAlis PRO* (Oxford Diffraction, 2012 ▶); cell refinement: *CrysAlis PRO*; data reduction: *CrysAlis PRO*; program(s) used to solve structure: *SHELXS97* (Sheldrick, 2008 ▶); program(s) used to refine structure: *SHELXL97* (Sheldrick, 2008 ▶); molecular graphics: *SHELXTL* (Sheldrick, 2008 ▶); software used to prepare material for publication: *SHELXTL*.

## Supplementary Material

Crystal structure: contains datablock(s) I, global. DOI: 10.1107/S1600536813003711/hy2615sup1.cif

Structure factors: contains datablock(s) I. DOI: 10.1107/S1600536813003711/hy2615Isup2.hkl

Additional supplementary materials:  crystallographic information; 3D view; checkCIF report

## Figures and Tables

**Fig. 1.**
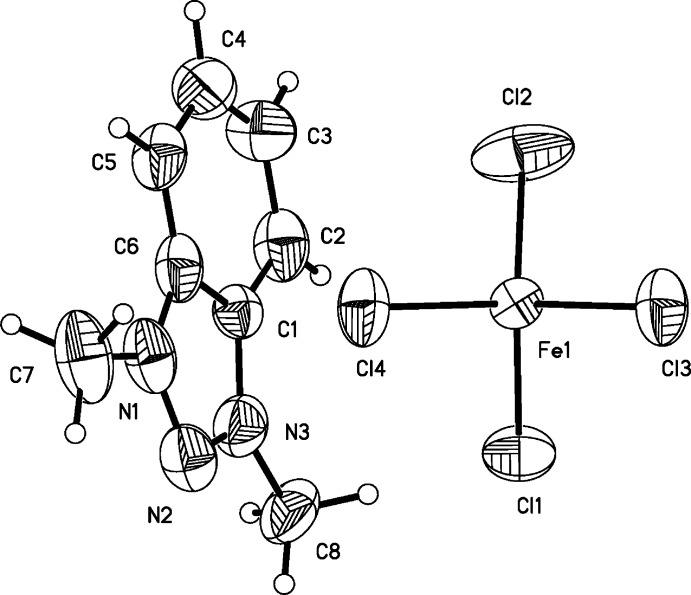
Molecular structure of the title compound. Displacement ellipsoids are drawn at the 50% probability level.

**Fig. 2.**
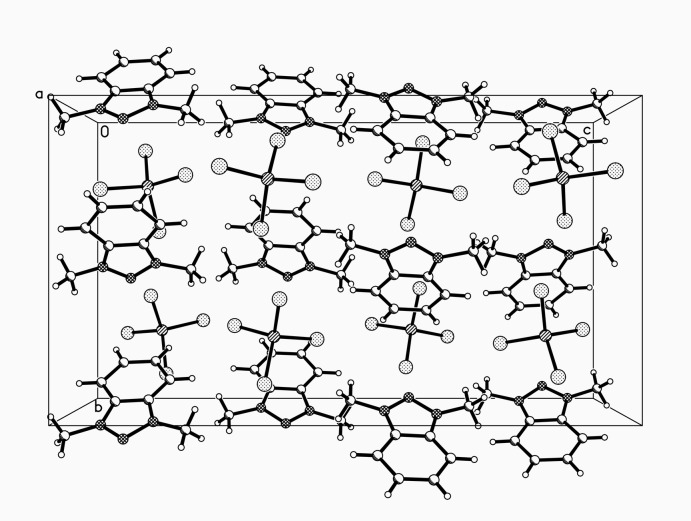
Crystal packing of the title compound viewed along the a axis.

## supplementary crystallographic information

### Comment

Iron-containing compounds are ubiquitous throughout the field of coordination chemistry. Recently, a variety of iron compounds have been added to the list of iron coordination complexes (Hay *et al.*, 2003; Liu *et al.*, 2000; Lorenz *et al.*, 2000; Shapley *et al.*, 2003).

In the title compound (Fig. 1), the Fe^III^ atom in the [FeCl_4_]^-^ anion is four-coordinated in a distorted tetrahedral geometry. The Cl—Fe—Cl bond angles are in the range of 108.35 (6)–111.33 (8) °, while the Fe—Cl bond lengths are in the range of 2.1634 (17)–2.1867 (14) Å. Three Cl—Fe—Cl angles are smaller than tetrahedral and the other three are greater than tetrahedral one. In the crystal, interactions between the Cl atoms and the triazolium rings are present [Cl···centroid distances = 3.587 (3) and 3.866 (3) Å].

### Experimental

FeCl_3_.6H_2_O (0.1 mmol, 27.0 mg) was dissolved in 15 ml CH_3_OH. To this yellow solution, one equivalent of 1,3-dimethyl-1*H*-benzo[1,2,3]triazolenium chlorate (0.1 mmol, 18.4 mg) was added with stirring. The mixture was filtered and held at room temperature to allow slow evaporation of solvent after stirring 30 min. Block crystals suitable for X-ray diffraction were obtained after one week (yield: 64%).

### Refinement

H atoms were positioned geometrically and refined as riding atoms, with C—H = 0.93 (CH) and 0.96 Å (CH_3_), and with *U*_iso_(H) = 1.5*U*_eq_(C) for methyl groups or 1.2*U*_eq_(C) otherwise.

### Crystal data

**Table:**

(C_8_H_10_N_3_)[FeCl_4_]	*F*(000) = 1384
*M**_r_* = 345.84	*D*_x_ = 1.575 Mg m^−^^3^
Orthorhombic, *P**b**c**a*	Mo *K*α radiation, λ = 0.71073 Å
Hall symbol: -P 2ac 2ab	Cell parameters from 1751 reflections
*a* = 10.2920 (5) Å	θ = 3.1–28.5°
*b* = 12.5518 (6) Å	µ = 1.74 mm^−^^1^
*c* = 22.5857 (9) Å	*T* = 293 K
*V* = 2917.7 (2) Å^3^	Block, yellow
*Z* = 8	0.32 × 0.28 × 0.25 mm

### Data collection

**Table:**

Oxford Diffraction SuperNova (Dual, Cu at zero, Eos) diffractometer	3016 independent reflections
Radiation source: fine-focus sealed tube	1828 reflections with *I* > 2σ(*I*)
Graphite monochromator	*R*_int_ = 0.031
Detector resolution: 16.0733 pixels mm^-1^	θ_max_ = 26.5°, θ_min_ = 3.1°
ω scans	*h* = −12→6
Absorption correction: multi-scan (*CrysAlis PRO*; Oxford Diffraction, 2012)	*k* = −15→12
*T*_min_ = 0.578, *T*_max_ = 0.647	*l* = −28→28
8114 measured reflections	

### Refinement

**Table:**

Refinement on *F*^2^	Primary atom site location: structure-invariant direct methods
Least-squares matrix: full	Secondary atom site location: difference Fourier map
*R*[*F*^2^ > 2σ(*F*^2^)] = 0.057	Hydrogen site location: inferred from neighbouring sites
*wR*(*F*^2^) = 0.183	H-atom parameters constrained
*S* = 1.05	*w* = 1/[σ^2^(*F*_o_^2^) + (0.0779*P*)^2^ + 1.072*P*] where *P* = (*F*_o_^2^ + 2*F*_c_^2^)/3
3016 reflections	(Δ/σ)_max_ < 0.001
147 parameters	Δρ_max_ = 0.53 e Å^−^^3^
0 restraints	Δρ_min_ = −0.39 e Å^−^^3^

### Special details

**Table:**

Geometry. All esds (except the esd in the dihedral angle between two l.s. planes) are estimated using the full covariance matrix. The cell esds are taken into account individually in the estimation of esds in distances, angles and torsion angles; correlations between esds in cell parameters are only used when they are defined by crystal symmetry. An approximate (isotropic) treatment of cell esds is used for estimating esds involving l.s. planes.
Refinement. Refinement of *F*^2^ against ALL reflections. The weighted *R*-factor *wR* and goodness of fit *S* are based on *F*^2^, conventional *R*-factors *R* are based on *F*, with *F* set to zero for negative *F*^2^. The threshold expression of *F*^2^ > σ(*F*^2^) is used only for calculating *R*-factors(gt) *etc*. and is not relevant to the choice of reflections for refinement. *R*-factors based on *F*^2^ are statistically about twice as large as those based on *F*, and *R*- factors based on ALL data will be even larger.

### Fractional atomic coordinates and isotropic or equivalent isotropic displacement parameters (Å^2^)

**Table:**

	*x*	*y*	*z*	*U*_iso_*/*U*_eq_	
N1	0.2130 (5)	0.5085 (4)	0.3377 (2)	0.0934 (14)	
N2	0.1550 (5)	0.5572 (4)	0.3863 (2)	0.0907 (14)	
N3	0.2259 (5)	0.5203 (4)	0.4309 (2)	0.0912 (13)	
C1	0.3237 (4)	0.4541 (4)	0.4130 (2)	0.0650 (11)	
C2	0.4177 (6)	0.3997 (5)	0.4452 (2)	0.0927 (17)	
H2	0.4238	0.4034	0.4863	0.111*	
C3	0.5016 (6)	0.3393 (5)	0.4106 (3)	0.0986 (17)	
H3	0.5669	0.3006	0.4293	0.118*	
C4	0.4922 (6)	0.3345 (6)	0.3510 (3)	0.1023 (18)	
H4	0.5522	0.2937	0.3302	0.123*	
C5	0.3950 (6)	0.3889 (5)	0.3193 (2)	0.0877 (16)	
H5	0.3874	0.3845	0.2783	0.105*	
C6	0.3140 (6)	0.4478 (5)	0.3524 (2)	0.0823 (15)	
C7	0.1582 (6)	0.5291 (6)	0.2780 (3)	0.124 (3)	
H7A	0.1317	0.4630	0.2604	0.186*	
H7B	0.2230	0.5622	0.2535	0.186*	
H7C	0.0844	0.5755	0.2814	0.186*	
C8	0.1916 (6)	0.5507 (5)	0.4913 (2)	0.111 (2)	
H8A	0.1229	0.6023	0.4903	0.166*	
H8B	0.2662	0.5808	0.5106	0.166*	
H8C	0.1632	0.4888	0.5127	0.166*	
Fe1	0.53140 (7)	0.75129 (5)	0.36820 (3)	0.0621 (3)	
Cl1	0.40700 (15)	0.76662 (12)	0.44622 (6)	0.0934 (5)	
Cl2	0.6707 (2)	0.62448 (18)	0.38165 (8)	0.1478 (9)	
Cl3	0.63300 (15)	0.90130 (13)	0.35368 (7)	0.0997 (5)	
Cl4	0.40818 (16)	0.71994 (13)	0.29142 (6)	0.1007 (5)	

### Atomic displacement parameters (Å^2^)

**Table:**

	*U*^11^	*U*^22^	*U*^33^	*U*^12^	*U*^13^	*U*^23^
N1	0.089 (3)	0.084 (3)	0.108 (4)	−0.025 (3)	−0.029 (3)	0.027 (3)
N2	0.086 (3)	0.079 (3)	0.107 (4)	−0.005 (3)	−0.020 (3)	0.016 (3)
N3	0.094 (3)	0.083 (3)	0.097 (3)	−0.019 (3)	0.010 (3)	−0.012 (3)
C1	0.064 (3)	0.070 (3)	0.060 (3)	−0.009 (2)	0.001 (2)	0.004 (2)
C2	0.105 (4)	0.108 (4)	0.064 (3)	−0.027 (4)	−0.012 (3)	0.008 (3)
C3	0.100 (4)	0.116 (5)	0.080 (4)	0.019 (4)	−0.001 (3)	0.007 (4)
C4	0.097 (4)	0.127 (5)	0.083 (4)	0.001 (4)	0.002 (3)	−0.012 (4)
C5	0.105 (4)	0.108 (4)	0.050 (3)	−0.030 (3)	0.009 (3)	−0.009 (3)
C6	0.089 (4)	0.087 (4)	0.071 (3)	−0.036 (3)	−0.003 (3)	0.003 (3)
C7	0.127 (5)	0.136 (6)	0.110 (5)	−0.039 (4)	−0.062 (4)	0.046 (4)
C8	0.130 (5)	0.109 (5)	0.093 (4)	−0.037 (4)	0.042 (4)	−0.043 (4)
Fe1	0.0725 (5)	0.0589 (5)	0.0548 (4)	0.0043 (3)	0.0006 (3)	0.0041 (3)
Cl1	0.1005 (10)	0.1047 (11)	0.0749 (9)	0.0156 (8)	0.0246 (8)	0.0135 (7)
Cl2	0.1852 (19)	0.1443 (18)	0.1138 (14)	0.1036 (16)	0.0155 (12)	0.0183 (11)
Cl3	0.1123 (11)	0.1022 (11)	0.0847 (10)	−0.0438 (9)	−0.0079 (8)	0.0060 (8)
Cl4	0.1173 (11)	0.1100 (11)	0.0748 (9)	−0.0393 (9)	−0.0193 (8)	0.0016 (8)

### Geometric parameters (Å, º)

**Table:**

N1—C6	1.332 (7)	C4—H4	0.9300
N1—N2	1.390 (7)	C5—C6	1.342 (7)
N1—C7	1.485 (7)	C5—H5	0.9300
N2—N3	1.327 (6)	C7—H7A	0.9600
N3—C1	1.367 (6)	C7—H7B	0.9600
N3—C8	1.460 (6)	C7—H7C	0.9600
C1—C6	1.373 (7)	C8—H8A	0.9600
C1—C2	1.390 (7)	C8—H8B	0.9600
C2—C3	1.390 (8)	C8—H8C	0.9600
C2—H2	0.9300	Fe1—Cl2	2.1636 (17)
C3—C4	1.351 (8)	Fe1—Cl3	2.1786 (15)
C3—H3	0.9300	Fe1—Cl4	2.1840 (14)
C4—C5	1.408 (8)	Fe1—Cl1	2.1867 (15)
			
C6—N1—N2	112.9 (5)	N1—C6—C5	131.3 (6)
C6—N1—C7	128.6 (6)	N1—C6—C1	105.8 (5)
N2—N1—C7	118.5 (5)	C5—C6—C1	122.9 (6)
N3—N2—N1	102.2 (4)	N1—C7—H7A	109.5
N2—N3—C1	113.1 (5)	N1—C7—H7B	109.5
N2—N3—C8	119.1 (5)	H7A—C7—H7B	109.5
C1—N3—C8	127.9 (5)	N1—C7—H7C	109.5
N3—C1—C6	106.1 (5)	H7A—C7—H7C	109.5
N3—C1—C2	131.0 (5)	H7B—C7—H7C	109.5
C6—C1—C2	122.9 (5)	N3—C8—H8A	109.5
C3—C2—C1	113.9 (5)	N3—C8—H8B	109.5
C3—C2—H2	123.0	H8A—C8—H8B	109.5
C1—C2—H2	123.0	N3—C8—H8C	109.5
C4—C3—C2	122.7 (5)	H8A—C8—H8C	109.5
C4—C3—H3	118.6	H8B—C8—H8C	109.5
C2—C3—H3	118.6	Cl2—Fe1—Cl3	109.81 (10)
C3—C4—C5	122.4 (6)	Cl2—Fe1—Cl4	111.32 (8)
C3—C4—H4	118.8	Cl3—Fe1—Cl4	108.36 (6)
C5—C4—H4	118.8	Cl2—Fe1—Cl1	109.85 (7)
C6—C5—C4	115.1 (5)	Cl3—Fe1—Cl1	109.04 (7)
C6—C5—H5	122.4	Cl4—Fe1—Cl1	108.41 (7)
C4—C5—H5	122.4		
			
C6—N1—N2—N3	−1.1 (5)	C3—C4—C5—C6	1.3 (8)
C7—N1—N2—N3	179.0 (4)	N2—N1—C6—C5	−179.6 (5)
N1—N2—N3—C1	0.9 (5)	C7—N1—C6—C5	0.4 (9)
N1—N2—N3—C8	−177.7 (4)	N2—N1—C6—C1	0.9 (5)
N2—N3—C1—C6	−0.4 (5)	C7—N1—C6—C1	−179.2 (5)
C8—N3—C1—C6	178.0 (5)	C4—C5—C6—N1	180.0 (5)
N2—N3—C1—C2	−179.8 (5)	C4—C5—C6—C1	−0.6 (7)
C8—N3—C1—C2	−1.4 (8)	N3—C1—C6—N1	−0.3 (5)
N3—C1—C2—C3	180.0 (5)	C2—C1—C6—N1	179.2 (5)
C6—C1—C2—C3	0.7 (7)	N3—C1—C6—C5	−179.9 (5)
C1—C2—C3—C4	0.1 (8)	C2—C1—C6—C5	−0.4 (7)
C2—C3—C4—C5	−1.1 (9)		
